# Supplementary material for: Chemical Profiling and Therapeutic Evaluation of Standardized Hydroalcoholic Extracts of Terminalia chebula Fruits Collected from Different Locations in Manipur against Colorectal Cancer
Source: Molecules. 2023 Mar 23;28(7):2901. doi: 10.3390/molecules28072901 (PMC10096451; doi:10.3390/molecules28072901)
Supplement: Supplementary file 1 [file molecules-28-02901-s001.zip › molecules-2244127-supplementary.pdf]

Supplementary Table S1: GCMS analysis of Hydroalcoholic extract of *T. chebula* fruits collected from Chandel, Manipur.

	RT	Compound	RSI	MW	Area %
1	20.29	1,2,3-Benzenetriol	917	126	20.95
2	40.04	2-Cyclopenten-1-one, 2-hydroxy-3,4-dimethyl	880	126	19.32
3	15.53	Catechol	924	110	12.82
4	16.23	5-Hydroxymethylfurfural	882	126	6.04
5	27.51	1-Hexyl-2-nitrocyclohexane	904	213	4.13
6	22.50	1-Undecanol	926	172	3.51
7	9.21	Phenol	914	94	3.27
8	40.12	1-Hexyl-2-nitrocyclohexane	972	213	2.3
9	36.98	Hexadecanamide	849	255	1.95
10	7.06	Isothiazole	818	85	1.93
11	13.79	4H-Pyran-4-one, 2,3-dihydro-3,5-dihydroxy-6- methyl	867	144	1.74
12	18.11	2,6-Difluorobenzoic acid, 4-nitrophenyl ester	856	279	1.72
13	10.19	1-(1'-pyrrolidinyl)-2-butanone	870	141	1.33
14	7.00	Ethanone, 1-(3-methyloxiranyl)-	837	100	0.98
15	8.98	Cyclobutaneethanol, á-methylene	916	112	0.92
16	10.84	Benzeneacetaldehyde	910	120	0.89
17	21.82	Sucrose	838	342	0.83
18	40.42	9-Octadecenamide	877	281	0.73
19	5.25	3-Furaldehyde	936	96	0.68
20	44.14	Naphthalene, 2-(1,1-dimethylethyl)decahydro -4a-methyl	852	208	0.66
21	27.66	Cyclotridecane	876	182	0.58
22	7.51	Pentanal, 2,2-dimethyl-	833	114	0.55
23	20.76	3-Methoxy-4-hydroxy mandelonitrile	830	179	0.55
24	9.60	2-Pyrrolidinecarboxylic acid, 1,2-dimethyl-5-oxo-, methyl ester	913	171	0.52
25	12.07	Furyl hydroxymethyl ketone	844	126	0.44
26	42.94	N-hydroxy-2,2,6,6-tetramethyl piperidin-4-one	882	171	0.44
27	17.61	Hydroquinone	849	110	0.43
28	25.83	Formic acid hydrazide	845	60	0.42
29	21.48	benzoic acid, 4-hydroxy-, 4-cyanophenyl ester	918	239	0.39
30	33.23	Cyclooctanemethanol, à,à-dimethyl	866	170	0.38
31	43.11	9H-Carbazole-1-carboxylic acid, 4-(1H-indol-3-yl)-, methyl ester	756	340	0.38
32	9.45	Phenol	865	94	0.34
33	8.60	2-Furancarboxaldehyde, 5-methyl	861	110	0.33
34	36.63	9-Octadecenamide	875	281	0.33
35	19.80	Pentyl glycolate	914	146	0.29
36	32.27	2,5-Cyclohexadien-1-one, 2,6-bis(1,1-dimethylethyl)-	883	232	0.28

		4-et hylidene			
37	9.56	2-Pyrrolidinecarboxylic acid, 1,2-dimethyl-5-oxo-, methyl ester	918	171	0.25
38	8.03	1H-Tetrazole, 5-vinyl-	860	96	0.23
39	21.97	Sucrose	823	342	0.22
40	44.77	8-Phenyl-6-thio-theophylline	759	272	0.22
41	13.57	3,5-Dimethyl-1,6-heptadien-4- ol	758	140	0.21
42	43.17	4-Methyl-2,4-bis(p-hydroxyphe nyl)pent-1-ene, 2TMS derivative	821	412	0.21
43	48.16	Purin-2,6-dione, 1,3-dimethyl-8-[2-[3,4-dimeth oxyphenyl]ethenyl]-	727	342	0.2
44	42.85	2H-1,3-Oxazine, tetrahydro-2,3,6-trimethyl	743	129	0.19
45	22.95	Ethyne, fluoro-	950	44	0.19
46	58.93	(7E)-2-Amino-4-hydroxy-7-[2- (4-methoxyphenyl)-2-oxoethyli dene]-7,8-dihydro-6(5H)-pteri dinone #	714	327	0.17
47	55.54	Phenol, 3-methoxy-5-methyl-2-(3,7,11 -trimethyl-2,6,10-dodecatrieny l)-, acetate, (E,E)-	648	384	0.17
48	58.83	Thieno[2,3-b]pyridine, 5-ethyl-3-nitro	698	208	0.16
49	33.93	Oxalic acid, isobutyl tetradecyl ester	830	342	0.15
50	25.34	1,2-Benzisothiazole, 3-butoxy-	813	207	0.15
51	49.92	3-Bromo-thiophene-2-carboxa mide	831	205	0.14
52	18.64	2-Buten-1-one, 1-(6,7,7-trimethyl-2,3-dioxabi cyclo[2.2.2]oct-5-en-1-yl)-, [1R-[1à(E),4á]]-	837	222	0.14
53	58.53	5-Benzofuranpropanol, 2-(1,3-benzodioxol-5-yl)-7-me thoxy	676	326	0.13
54	17.00	1-Hexyl-2-nitrocyclohexane	895	213	0.13
55	6.38	Cyclopropylacetylene	870	66	0.13
56	58.45	Cyclododecaneacetaldehyde	702	210	0.12
57	57.16	1-Formyl-2,6-dimethoxy-10-m ethyl-anthracene	742	280	0.12
58	54.14	2-Formamidobenzophenone	864	225	0.12
59	51.08	1,4-Dihydro-3-(4-methoxyphen yl)-1,7-dimethyl-6H-[1,2,4]tri azino[4,3-b][1,2,4,5]tetrazin-6 -one	748	286	0.12
60	15.24	Hexanoic acid, 5-methyl-3-oxo-, methyl ester	878	158	0.12
61	6.51	1H-Imidazole-2-carboxaldehy de	802	96	0.12
62	52.86	Furan, 2,2'-[1,2-ethanediylbis(oxy)]bi s[tetrahydro-5-(2-methoxy-4- methylphenyl)-5-methyl	768	470	0.11
63	50.92	1,2,3,4-Tetrahydro-1-methyl-7 -(4-nitrophenyl)oxazolo[2,3-f]p urine-2,4-dione	710	327	0.11
64	47.07	Pyridine, 1-acetyl-5-(3,4-dihydro-2H-py rrol-5-yl)-1,2,3,4-tetrahydro	766	192	0.11
65	38.26	Cyclopentadecanone, oxime	792	239	0.11
66	28.76	1H-1,2,3-Triazole-4-carboxald ehyde	838	97	0.11

67	17.31	Silane, 2-butenyltrifluoro-	880	140	0.11
68	17.14	Thiophen-2-methylamine, N-decyl-N-methyl	865	267	0.11
69	21.06	3-Penten-1-yne, 3-methyl-	838	80	0.1
70	13.41	Cyclohexene, 3-(2,2-dimethylpropoxy)-	781	168	0.1
71	12.93	Ethyne, fluoro-	978	44	0.1

Supplementary Table S2: GCMS analysis of Hydroalcoholic extract of *T. chebula* fruits collected from Kakching, Manipur.

	RT	Compound	RSI	MW	Area %
1	20.29	1,2,3-Benzenetriol	920	126	26.53
2	40.03	2-Cyclopenten-1-one, 2-hydroxy-3,4-dimethyl	877	126	12.79
3	16.23	5-Hydroxymethylfurfural	891	126	11.26
4	15.55	Catechol	925	110	6.85
5	9.22	Phosphonic acid, (p-hydroxyphenyl)-	914	174	3.82
6	18.11	2,6-Difluorobenzoic acid, 4-nitrophenyl ester	865	279	3.05
7	27.51	1-Hexyl-2-nitrocyclohexane	906	213	2.76
8	21.83	Sucrose	820	342	2.27
9	22.51	1-Undecanol	922	172	1.88
10	13.80	4H-Pyran-4-one, 2,3-dihydro-3,5-dihydroxy-6- methyl	856	144	1.68
11	5.25	3-Furaldehyde	947	96	1.59
12	10.20	2-Cyclohexen-1-one	807	96	1.49
13	8.99	Cyclobutaneethanol, á-methylene	903	112	1.4
14	36.98	Hexadecanamide	841	255	1.31
15	20.76	sedanolide	887	194	1.14
16	10.85	Benzeneacetaldehyde	899	120	1.13
17	19.78	Pentyl glycolate	855	146	0.99
18	44.13	1-Methyl-4-isopropyl-cyclohe xyl 2-hydroperfluorobutanoate	917	334	0.85
19	44.76	9H-Xanthen-9-one, 1,3-dihydroxy-6-methoxy-8-me thyl	839	272	0.72
20	8.03	Cyclopropene	899	40	0.67
21	12.08	2-Heptanone, 6-methyl-5-methylene	899	140	0.67
22	40.42	Cyclooctanemethanol, à,à-dimethyl	836	170	0.61
23	20.11	1-Phenyl-1-decanol	897	234	0.6
24	18.22	(E)-4-Hydroxy-2-methyl-pent-2 -enoic acid	880	130	0.52
25	9.63	Carbamic acid, phenyl ester	838	137	0.45
26	25.78	Cyclohexane, 1,1'-[1,2-bis(1,1-dimethylethyl)-1,2-ethanediyl]bis-, (R*,R*)-(ñ)-	769	306	0.44
27	21.20	3,6-methanonaphth[2,3-b]oxire ne-2,7-dione, octahydro	844	192	0.43

28	8.61	2-Furancarboxaldehyde, 5-methyl	831	110	0.42
29	9.46	Phenol	861	94	0.37
30	22.87	Nitrous acid, ethyl ester	971	75	0.37
31	27.67	1-Hexadecanol	861	242	0.36
32	7.58	Proline, 2-methyl-5-oxo-, methyl ester	964	157	0.34
33	12.66	1,3-Dioxolan-2-one, 4,5-bis(methylene)-	869	112	0.34
34	19.92	Propanoic acid, 3-chloro-, 4-formylphenyl ester	831	212	0.34
35	26.95	2,4(1H,3H)-Quinolinedione, 3-benzoyl-3-(phenylmethyl)-	858	355	0.3
36	10.44	2,4-Azetidinedione, 3,3-diethyl-1-methyl	837	155	0.29
37	21.06	2-Methyl-1,3-oxazole-4-carbo nitrile	825	108	0.28
38	20.68	2,2-Diethyl-N-ethylpyrrolidine	839	155	0.27
39	5.51	2-Pentanone, 4-hydroxy-4-methyl	810	116	0.26
40	25.87	2-Methyl-1,3-oxathiolane-2-a cetic acid methyl ester	833	176	0.26
41	36.64	4,4-dimethyl-5-oxo-tetrahydro furan-3-carboxylic acid	841	158	0.26
42	13.57	Methyl 2-[methoxy(methyl)amino]-2- methylpropanoate	844		0.24
43	6.40	1,3-Cyclopentadiene	861	66	0.23
44	9.58	Carbamic acid, methyl-, phenyl ester	855	151	0.23
45	21.48	(9S,10R)-9,10-Epoxy-3Z,6Z-h eneicosadiene	864	306	0.23
46	33.23	Cyclooctanemethanol, à,à-dimethyl	851	170	0.23
47	10.72	2-Butyne	925	54	0.22
48	22.26	Disulfide, methyl (methylthio)phenylmethyl	873	216	0.2
49	48.16	1,3,5-Trioxepane	875	104	0.19
50	43.23	1,2,4-Benzenetricarboxylic acid, 1,2-dimethyl ester	800	238	0.19
51	17.51	4-Aminophenyl trifluoromethanesulfonate	813	241	0.19
52	17.14	1,2-Benzenediol, 3-methoxy-	807	140	0.19
53	32.27	2,5-Cyclohexadien-1-one, 2,6-bis(1,1-dimethylethyl)-4-et hylidene	846	232	0.18
54	56.59	11,12-Dihydroxyseychellane	797	238	0.17
55	43.29	2-Cyclohexen-1-one, 3-[2-(acetyloxy)butyl]-2,4,4-tr imethyl	846	252	0.17
56	6.53	4-Cyclopentene-1,3-dione	822	96	0.17
57	48.41	5-Bromo-thiophene-2-carboxa mide	742	205	0.16
58	13.41	1H-Tetrazol-5-amine	772	85	0.15
59	46.98	1H-Indene, 2,4,5,6,7,7a-hexahydro-7a-me thyl-3-(2-methylpropyl)-	809	192	0.14
60	33.93	2-Hexyl-1-octanol	866	214	0.14
61	20.89	3-Hexen-1-yne	842	80	0.14
62	38.26	2-Hexanone, 3-hydroxy-3,5-dimethyl	775	144	0.13
63	5.84	Ethyne, fluoro-	961	44	0.13

64	15.24	2-tert-Butyl-3,4,5,6-tetrahydro pyridine	830	139	0.12

Supplementary Table S3: GCMS analysis of Hydroalcoholic extract of *T. chebula* fruits collected from Sagolband, Manipur.

	RT	Compound	RSI	MW	Area %
1	20.30	1,2,3-Benzenetriol	921	126	40.41
2	40.04	2-Cyclopenten-1-one, 2-hydroxy-3,4-dimethyl	874	126	14.66
3	15.53	Catechol	929	110	12.47
4	9.21	Phosphonic acid, (p-hydroxyphenyl)-	937	174	4.64
5	27.51	1-Hexyl-2-nitrocyclohexane	908	213	2.11
6	22.50	1-Undecanol	927	172	1.74
7	36.98	Hexadecanamide	860	255	1.56
8	25.73	S-Methyl 2-methylpropanethioate	852	118	1.26
9	10.18	2-Cyclohexen-1-one	838	96	1.22
10	16.31	Diazene, 1-cyclopentyl-2-methoxy-, 1-oxide	818	144	0.94
11	18.11	2,4-Difluorobenzoic acid, 4-nitrophenyl ester	875	279	0.88
12	8.98	2-Butenal, 3-methyl-	892	84	0.85
13	13.81	4H-Pyran-4-one, 2,3-dihydro-3,5-dihydroxy-6- methyl	853	144	0.74
14	20.77	Vanillin	812	152	0.74
15	7.08	Ethanone, 1-(3-methyloxiranyl)-	841	100	0.71
16	19.81	Pentyl glycolate	902	146	0.70
17	9.56	1,2-Cyclohexanedione	833	112	0.69
18	16.25	4-Ethyl-2-hydroxycyclopent-2- en-1-one	819	126	0.58
19	5.27	3-Furaldehyde	930	96	0.50
20	40.43	9-Octadecenamide	852	281	0.49
21	22.95	Nitrous acid, ethyl ester	986	75	0.47
22	9.45	Cyclopropylacetylene	883	66	0.44
23	44.76	1,1'-Diethyl-2,3,2',3'-tetrahydr o-2,2'-diperimidine	877	394	0.43
24	8.60	2-Furancarboxaldehyde, 5-methyl	879	110	0.40
25	10.85	Cyclobutene, 2-propenylidene-	892	92	0.38
26	21.48	4-Hydroxy-à-bromoethylpheno ne	903	228	0.38
27	7.02	Ethanone, 1-(3-methyloxiranyl)-	848	100	0.37
28	36.64	Oxacyclohexadecan-2-one, 16-methyl	895	254	0.29
29	33.23	1,8-Nonanediol, 8-methyl-	843	174	0.28
30	27.67	Cetene	871	224	0.27
31	17.62	1,2,4,5-Tetrazine	888	82	0.25
32	21.07	1,2,3-Benzenetriol	856	126	0.24

33	12.66	1,3-Dioxolan-2-one, 4,5-bis(methylene)-	893	112	0.23
34	6.39	1,3-Cyclopentadiene	887	66	0.20
35	43.10	2-(14-Carboxytetradecyl)-2-eth yl-4,4-dimethyl-1,3-oxazolidin e-N-oxyl	782	384	0.19
36	33.07	Phthalic acid, butyl hept-4-yl ester	908	320	0.19
37	6.52	4-Cyclopentene-1,3-dione	843	96	0.19
38	32.27	Tetradecanoic acid, 10,13-dimethyl-, methyl ester	913	270	0.18
39	17.14	4-Heptanone, 1,1,1,7,7,7-hexafluoro	804	222	0.18
40	8.03	2,5-Furandione, 3-methyl-	874	112	0.18
41	42.95	2-Propanone, hydrazone	752	72	0.17
42	21.23	Methylpyrazine-2-carboxylate	796	138	0.17
43	40.69	4-Cycloocten-1-one, 8-butyl-, oxime, (Z,Z)-	902	195	0.16
44	10.47	2H-Pyran-2,6(3H)-dione	811	112	0.16
45	7.53	Pentanal, 2,2-dimethyl-	799	114	0.16
46	45.36	1,1,1,3,5,5,7,7,7-Nonamethyl-3 -(trimethylsiloxy)tetrasiloxane	773	384	0.15
47	39.95	5,10-Pentadecadienoic acid, (E,Z)-	910	238	0.15
48	12.97	Butanoic acid, 4-hydroxy-2-methylene	822	116	0.15
49	36.06	1,1,1,3,5,5,7,7,7-Nonamethyl-3 -(trimethylsiloxy)tetrasiloxane	843	384	0.14
50	22.27	9-Azabicyclo[3.3.1]non-2-ene9-carboxylic acid, 6-(acetyloxy)-, ethyl ester, endo	852	253	0.14
51	43.26	Capsaicin, TMS derivative	824	377	0.13
52	21.97	2-Ethoxyamphetamine	882	179	0.13
53	11.52	Ethanone, 1-(1H-pyrrol-2-yl)-	787	109	0.13
54	24.57	Ethyne, fluoro	955	44	0.12
55	10.72	2-Butyne	918	54	0.12
56	33.94	1-Octanol, 2-butyl-	870	186	0.11
57	25.33	3-Methoxyformanilide	934	151	0.11
58	51.55	Nalbuphine, dimethyl deriv	796	385	0.10
59	43.20	4-(4-Hydroxyphenyl)-4-methy l-2-pentanone, TMS derivative	887	264	0.10

Supplementary Table S4: GCMS analysis of Hydroalcoholic extract of *T chebula* fruits collected from Yumnam Huidrom, Manipur.

	RT	Compound name	RSI	Molecular weight	Area %
1	20.34	1,2,3-Benzenetriol	930	126	43.56
2	40.07	2-Hydroxy-3,5-dimethylcyclop ent-2-en-1-one	914	126	9.07
3	9.27	Phenol	915	94	5.40

4	16.32	5-Hydroxymethylfurfural	888	126	4.03
5	10.26	2,3,4,5-Tetrahydropyridazine	868	84	1.65
6	15.62	1,2-Benzenediol, mono(methylcarbamate)	905	167	1.57
7	10.89	Benzeneacetaldehyde	890	120	1.56
8	8.07	2,5-Furandione, 3-methyl-	869	112	1.18
9	25.83	2,6,10,14-Tetramethylpentade can-6-ol	848	284	1.06
10	37.02	3-Ethyl-2-methyl-2-heptanol	849	158	0.99
11	9.06	Cyclobutaneethanol, á-methylene	880	112	0.93
12	15.75	1,2-Benzenediol, mono(methylcarbamate)	879	167	0.91
13	5.33	3-Furaldehyde	896	96	0.89
14	17.17	Furan, 2-(1,2-diethoxyethyl)-	879	184	0.77
15	22.64	1,2-Dicarboxy-3-(4-chlorophe nyl)-2,3(1H)-dihydropyrido(1, 2-a)benzimidazole	887	370	0.73
16	22.94	3,4,5-Trimethoxy-á-methyl-á-n itrostyrene	727	253	0.71
17	24.09	2-Butenedioic acid, dibutyl ester	873	228	0.70
18	12.70	Hex-4-yn-3-one	822	96	0.68
19	10.77	1-Methylcyclopropene	866	54	0.64
20	21.52	4-Hydroxy-à-bromoethylpheno ne	898	228	0.64
21	25.71	1H-1,3-Diazepine, 4,5,6,7-tetrahydro-2-methyl	843	112	0.64
22	32.31	2,5-Cyclohexadien-1-one, 2,6-bis(1,1-dimethylethyl)-4-et hylidene	882	232	0.64
23	20.81	3-Methoxy-4-hydroxy mandelonitrile	820	179	0.63
24	9.48	2,2,4-Trimethyl-3-pentanone	845	128	0.59
25	53.99	Glycinamide, N(2)-methyl-	711	88	0.59
26	22.01	1-Phenanthrenecarboxylic acid	668	346	0.54
27	23.55	6-Fluoro-2-trifluoromethylben zoic acid, 2-formyl-4,6-dichlorophenyl ester	893	380	0.51
28	57.92	(7E)-2-Amino-4-hydroxy-7-[2- (4-methoxyphenyl)-2-oxoethyli dene]-7,8-dihydro-6(5H)-pteri dinone #	688	327	0.50
29	17.35	Ritalinic acid	676	219	0.47
30	43.34	Formamide, N,N-dimethyl-	828	73	0.47
31	36.69	Phosphonofluoridothioic hydrazide, P,2,2-trimethyl	789	156	0.45
32	9.68	2-Ethyl-5-propylcyclopentano ne	819	154	0.44
33	25.05	5,6,8,4'-Tetrahydroxy-7,3'-dim ethoxyflavone	778	346	0.42
34	54.87	Thieno[2,3-b]pyridine, 5-ethyl-3-nitro	817	208	0.39
35	12.16	4-Methyl-2,4-bis(p-hydroxyphe nyl)pent-1-ene, 2TMS derivative	710	412	0.37
36	44.83	Ferrocenecarboxylic acid, 1',2-dimethyl-,	790	272	0.37

		methyl ester			
37	48.69	16-Azatricyclo[9.2.2.1(4,8])he xadeca-4,6,8(16),11,13,14-hex aene, 16-oxide	823	225	0.37
38	8.68	2-Furancarboxaldehyde, 5-methyl	873	110	0.36
39	36.19	9 1-(2-(3-Cyclohexenyl)ethyl)sila trane	925	283	0.36
40	50.92	4-(Trimethylsilyl)pyrazole, 2TMS derivative	724	212	0.36
41	44.28	2,2-Diphenyl-6-methyl-1,3-di oxa-6-aza-2-silacyclooctane	739	299	0.35
42	45.62	4-Methyl-2H-pyran	751	96	0.35
43	62.10	Urea, propyl-	802	102	0.35
44	24.59	Propanamide, N-(aminocarbonyl)-	837	116	0.34
45	52.34	Benzene, 5-(2-isothiocyanatoethyl)-1,2, 3-trimethoxy	716	253	0.34
46	52.07	4-(4-Hydroxyphenyl)-4-methy l-2-pentanone, TMS derivative	879	264	0.33
47	59.64	1,2,3-Triphenyl-3-isopropyl-c yclopropene	789	310	0.33
48	43.23	2-Butanol, 3-methyl-4-(trimethylstannyl)-	796	252	0.32
49	48.21	Ethyl 2-methyl-1,3-benzodioxole-2-e thanoate	777	222	0.32
50	50.12	Phenanthrene, 4-methoxy-	800	208	0.32
51	50.42	1,2,3,4-Tetrahydro-1-methyl-7 -(4-nitrophenyl)oxazolo[2,3-f]p urine-2,4-dione	772	327	0.32
52	21.10	3-Hexen-1-yne	860	80	0.31
53	44.45	Thieno[2,3-b]pyridine, 5-ethyl-3-nitro	738	208	0.31
54	48.92	2,3,4,5-Tetrahydro-7,8-(methy lenedioxy)-N-(diethylphospho ryl)-3-benzazepin-1-one	813	341	0.30
55	56.15	Pyrido[3,2-d]pyrimidin-4-ol	770	147	0.30
56	20.05	1-Phenanthrenecarboxylic acid,	809	346	0.29
57	47.66	2,2-Dimethyl-7-methoxy-chrom anone	809	206	0.29
58	43.29	1-Methyl-1,2,2-tri(pentafluoro benzoyl)-hydrazine	788	628	0.28
59	52.50	3(2H)-Benzofuranone, 6-methoxy-2-[(3-methoxyphe nyl)methylene]-, (E)-	775	282	0.28
60	25.92	1-Phenanthrenecarboxylic acid,	788	346	0.27
61	43.45	Thiophen-2-methylamine, N-(2-fluorophenyl)-	732	207	0.27
62	60.56	Bismuthine, triethyl	722	296	0.27
63	52.90	1,3-Dioxolo[4,5-c]acridin-6(11 H)-one, 5-ethoxy-4-methoxy-11-methy l	781	327	0.26

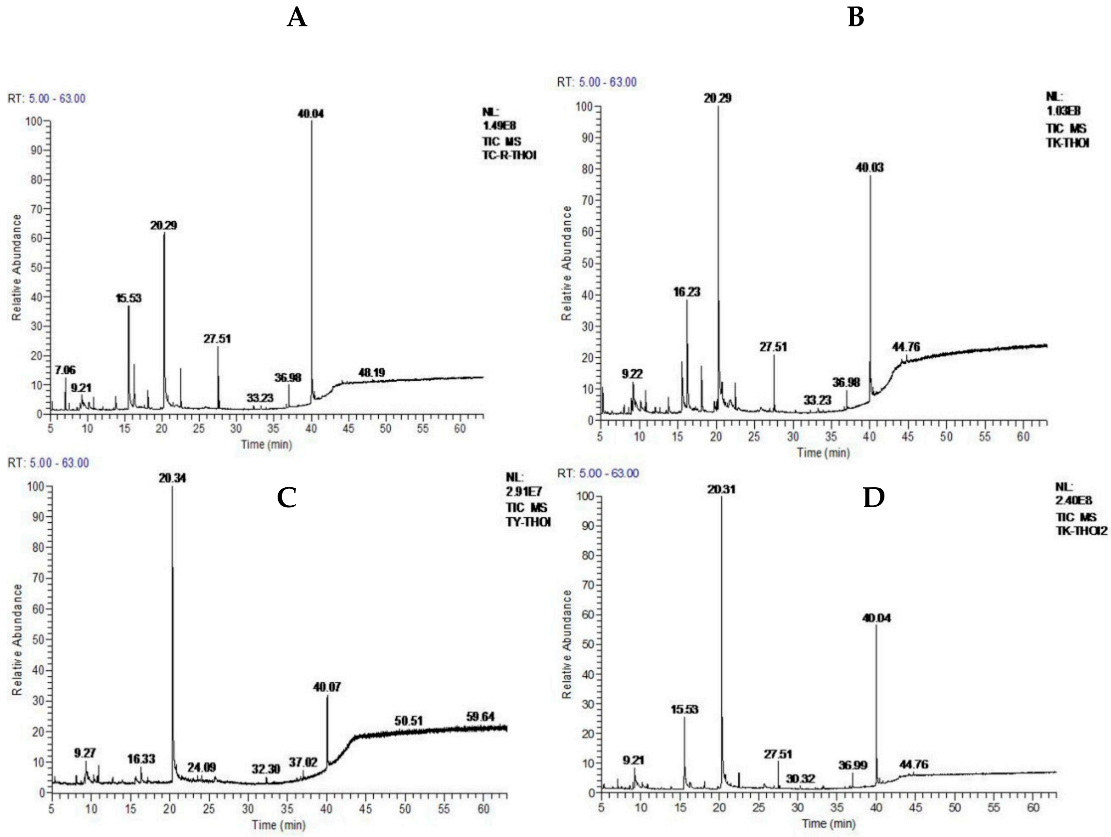

Supplementary Figure S1: GCMS profile of hydroalcoholic extract of *T. chebula* fruits collected from four (4) different locations of Manipur. (**A**) Hydroalcoholic extract of *T. chebula* collected from Chandel, Manipur. (**B**) Hydroalcoholic extract of *T. chebula* collected from Kakching, Manipur. (**C**) Hydroalcoholic extract of *T. chebula* collected from Yumnam Huidrom, Manipur (D) Hydroalcoholic extract of *T. chebula* collected from, Manipur.

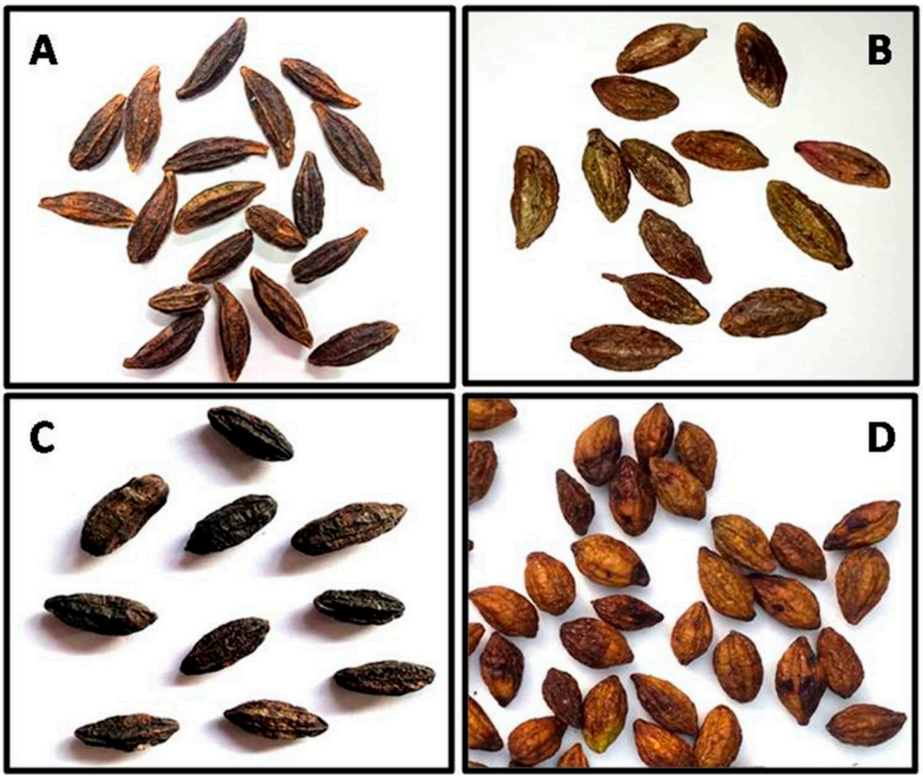

Supplementary Figure S2: Fruits of *T chebula* collected from Chandel, Manipur (**TCH**); (**B**) Kakching, Manipur (**TKH**); (**C**) Sagolband, Imphal East, Manipur (**TSH**) and Yumnam Huidrom, Manipur (**TYH**).

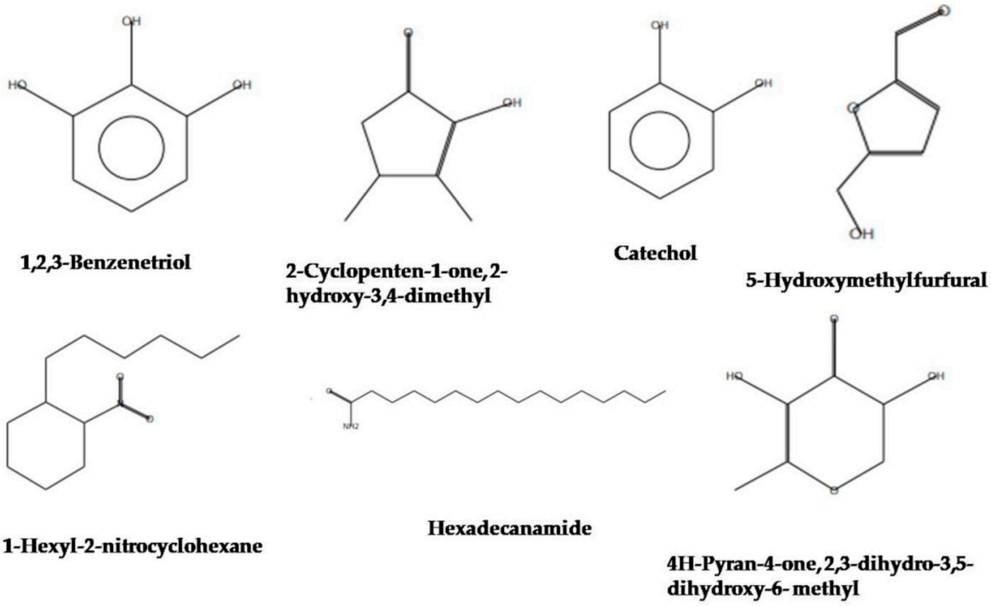

Supplementary Figure S3: Structural representation of the major compounds detected in GC-MS results of the *T chebula* fruit extracts
